# A *Trichinella spiralis* new born larvae-specific protein, *Ts-*NBL1, interacts with host’s cell vimentin

**DOI:** 10.1007/s00436-022-07479-7

**Published:** 2022-03-23

**Authors:** A. Wang, X. Liu, A. Heckmann, G. Caignard, D. Vitour, E. Hirchaud, M. Liu, P. Boireau, G. Karadjian, I. Vallée

**Affiliations:** 1 UMR BIPAR, Anses, Laboratoire de Santé Animale, INRAE, Ecole Nationale Vétérinaire d’Alfort, Maisons-Alfort, France; 2 Key Laboratory of Zoonosis Research, Ministry of Education, Institute of Zoonosis, College of Veterinary Medicine, Jilin University, Changchun, China; 3 UMR 1161 Virologie, ANSES, Laboratoire de Santé Animale, INRAE, Ecole Nationale Vétérinaire d’Alfort, Paris‑Est Sup, Maisons‑Alfort, France; 4 Viral Genetic and Biosecurity Unit, BP53, ANSES Ploufragan-Plouzané-Niort Laboratory, Ploufragan, France

**Keywords:** *Trichinella*, Vimentin, Proteins interaction

## Abstract

The parasitic nematode *Trichinella* has a special relationship with its host as it has a unique intracellular location within the feeder cell which is a structure derived from skeletal muscle fiber. It has been proposed that “parakines” secreted by *Trichinella* larvae serve as messengers to implement communication between the parasite and the muscle cells through a molecular cross-talk to ensure permanent coexistence within the host. The *Ts-*NBL1 protein is considered to be a potential key “parakine” involved in the early invasion of the muscle fiber and its transformation into a feeder cell during *Trichinella spiralis* infection. This study used for the first time yeast two-hybrid (Y2H) technology in *Trichinella* to identify *Ts-*NBL1 interacting proteins. GST co-affinity purification experiments confirmed vimentin as an important interactor. The discovery of the new host proteins interacting with *Ts-*NBL1 will help to suggest that *Ts-*NBL1 contributes to participate in the capsule formation of feeder cells and provide ideas for understanding the molecular and cellular mechanisms involved in the survival of *Trichinella* in the host.

**Supplementary Information:**

The online version contains supplementary material available at 10.1007/s00436-022-07479-7.

## Introduction

*Trichinella* is a genus of intracellular parasitic nematodes distributed worldwide, with the ability to infect almost all monogastric mammals including humans, birds, and reptiles. It was ranked third among the 25 most significant foodborne parasitic diseases at the European level (Bouwknegt et al. 2018). *Trichinella* genus is divided in two clades. While the species of both clades are capable of forming a feeding site named “nurse cell” (NC), they can be distinguished by the collagen capsule that surrounds it. Members of one clade produce a typical collagen-based cyst wall and are called encapsulated species. On the other hand, the collagen capsule of the other clade is poorly developed and difficult to visualize under the light microscope; therefore, it is referred to a non-encapsulated species (Xu et al. 1997).

The life cycle of *T. spiralis* is completed within a single host species and infection starts with the ingestion of infective muscle larvae followed by digestion of the protective capsule within the host stomach (Gottstein et al. 2009). The muscle larvae migrate to the small intestine where they penetrate intestinal epithelium and undergo four molts over a 30-h period to reach sexual maturity. The adults mate within the intestinal epithelium, and embryogenesis takes on 4 days post-infection (dpi). Each female releases newborn larvae (NBL) approximately 4–6 dpi (Gagliardo et al. 2002). The NBL exit the epithelium from the basolateral face, migrate through the bloodstream and lymphatic vessels, and then invade the cells of the striated muscles. The major secretory cells of the parasites, the stichocytes, increase in number from about 20 on day 1 to about 50–55 by day 19 (Despommier and Muller 1976). NBL infection of striated muscle cells induces developmental changes culminating with the formation of a novel host-parasite structure known as “nurse cell.” This is composed of a collagenous wall and cellular components that contribute to the protection of the parasite from its host immune response and participate in its long-term survival. The development of the NC is complex and it is believed that some factors produced by *Trichinella*, called “parakines,” may act as messengers to communicate with the host at the molecular level and ultimately alter the host cells’ fate to meet the parasite’s needs (Despommier 1998). Although the existence of “parakines” has been predicted on the basis on the complex interactions that occur between the mammalian cell and the parasite during NC formation, no putative “parakine” has been characterized so far. Little is known about the molecular and cellular factors and their mechanisms involved in *Trichinella* development and its survival within the cytoplasm of the NC. However, since mRNA coding for collagen is present within the NC at 9 dpi, studies have suggested that NC formation mainly begins during the NBL stage from 4 to 15 dpi (Polvere et al. 1997; Despommier 1998). Therefore, more attention should be paid on the early stage of *Trichinella* infection, as striated muscle fibers might receive parasitic signals before their invasion by NBL.

Multiple proteases have been reported in excretory/secretory products from parasitic nematodes. Their potential roles is to penetrate the host tissues, molt, and evade host innate defenses (Hewitson et al. 2009; Yang et al. 2015, p. 1; Abuzeid et al. 2020). Most of *Trichinella spiralis* proteases such as TsSerP (Trap et al. 2006) or TspSP-1.2 (Wang et al. 2013, p. 5) are excreted at all stages, but a few are stage specific such as TspSP1, excreted by the muscle larvae stichocytes (Romaris et al. 2002). Interestingly, *Trichinella spiralis* NBL1 (*Ts-*NBL1) was the first identified early stage newborn larvae-specific protein of *T. spiralis* (Liu et al. 2007). It has a signal peptide at the N-terminus which could destinate the protein toward the secretory pathway, a conserved trypsin-like domain, and its C-terminal part is highly immunogenic (Yang et al. 2015, 2016). The early-stage-specific and secreted characteristics make *Ts*-NBL1 a potential key factor involved in *Trichinella* invasion and the development of nurse cell.

The aim of this study was indeed to analyze whether or not *Ts-*NBL1 could be linked to any interaction with cell factors particularly in the formation of the collagen capsule by checking first its belonging to the encapsulated clade or not. Our work was focused on *Ts-*NBL1 interactions with the host’s proteins by using yeast two-hybrid system which is widely used for screening of protein–protein interactions (Fields and Song 1989). Interaction of *Ts-*NBL1 with identified protein was confirmed with GST pull-down, qPCR, and co-localization by immunofluorescence labelling.

## Materials and methods

### Phylogenic analysis and sequence alignments of *Ts-*NBL1 with other species of the *Trichinella* genus

A homology gene analysis of *Ts-*NBL1, *T. spiralis* (AAR36900.1), with other *Trichinella* species was performed with sequences from *T*. *nativa* (KRZ256488.1), *T*. *britovi* (KRY56140.1), *T*. *nelsoni* (KRX20959.1), *T. murrelli* (KRX46800.1), T6 (KRX76874.1), T8 (KRZ92112.1), T9 (KRX65963.1), *T. patagoniensis* (KRY14817.1), *T*. *pseudospiralis* (AEV21546.1), *T. papuae* (KRZ79292.1), and *T*. *zimbabwensis* (KRZ08368.1). MEGAX and ClustalX programs were used for the construction of phylogenetic tree and the comparison of multiple sequence alignments.

### Cloning

**Fig. 1:**
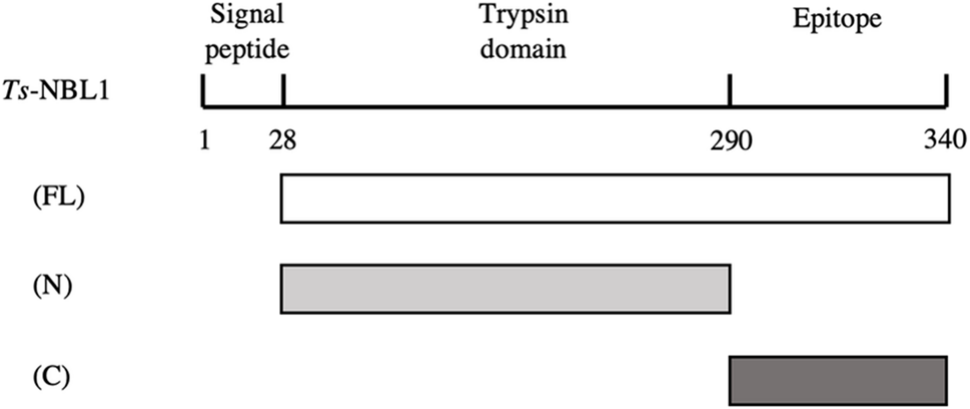
Construction of the *Ts-NBL1* plasmid. Schematic illustration of *Trichinella spiralis NBL1* fragments. The signal peptide is from the first to the 28th amino acid (AA), and the full length (FL) of *Ts-NBL1* is from the 28th to the 340th AA. It is composed of the N-terminal part (N) containing the trypsin domain (from AA 28 to AA 290) and of the C-terminal part (C) which contains the immuno-dominant sequence (from AA 290 to AA 340)

GenBank accession number *Ts-*NBL1 (AY_491941.1) and vimentin (NM_003380) coding sequence were amplified by RT-PCR from total RNAs extracted from *T. spiralis* (ISS_004) newborn larvae and human foreskin fibroblast (HFF) cells which were stored in − 80 ℃, respectively. DNA sequences encoding *Ts-NBL1*-FL (full length), *Ts-NBL1*-N (N-terminal, AA 29–290), *Ts-NBL1*-C (C-terminal, AA 291–430) (Fig. 1), and vimentin were amplified with Gateway cloning primers, 5′ ends of forward primers were fused to attB1.1 recombination sequence 5′-GGGGACAACTTTGTACAAAAAAGTTGGCATG-3′, while reverse primers were fused to attB2.1 recombination sequence 5′-GGGGACAACTTTGTACAAGAAAGTTGGTTA-3′ (Table 1). Standard PCR was performed and PCR products were cloned into pDONR207 by an in vitro recombination-based cloning system (BP reaction, Gateway system, Invitrogen) following manufacturer’s recommendation. *Ts-NBL1* fragments or vimentin encoding sequences were transferred by an in vitro recombination (LR reaction) from pDONR207 into pmCherry-C1, pCIneo**-**3xFlag, or pDEST27-GST (Caignard et al. 2013) following manufacturer’s recommendation. All recombinant constructions were transformed and amplified in *Escherichia coli* DH5α strain (Invitrogen).

**Table 1:** Primer sequences for Gateway reactions with attB sites

*Ts-NBL1-*FL	Fwd 5′-3′	GGGGACAACTTTGTACAAAAAAGTTGGCATGGCG TTTGAATGCGGTGTGCCACATTTTAA
	Rev 5′-3′	GGGGACAACTTTGTACAAGAAAGTTGGTTACTTAG AAAAGTGATAATATGTTG
*Ts-NBL1-*N	Rev 5′-3′	GGGGACAACTTTGTACAAGAAAGTTGGTTACTTAG AAAAGTGATAATATGTTG
*Ts-NBL1-*C	Fwd 5′-3′	GGGGACAACTTTGTACAAAAAAGTTGGCGAAAATT CTCCTGAAGGAACTG
*Vimentin*	Fwd 5′-3′	GGGGACAACTTTGTACAAAAAAGTTGGCATGTCCA CCAGGTCCGTGTCCTCG
	Rev 5′-3′	GGGGACAACTTTGTACAAGAAAGTTGGTTATTCAA GGTCATCGTGATGCTG

### Cell culture and transient transfection

All cells were maintained in Dulbecco’s modified Eagle’s medium (DMEM, Invitrogen) supplemented with 10% heat inactivated fetal bovine serum, penicillin, and streptomycin at 37 ℃ with 5% CO_2_. C2C12 cells (ATCC® CRL-1772™) were dispensed in 12-well plates 24 h before use at 1 × 10^5^ cells/cm^2^ in order to obtain a supportive monolayer for stem cell growth. Cherry alone and recombinant Cherry-*Ts-NBL1* vectors were transfected to C2C12 cells at 1 µg of each plasmid DNA per well. To achieve overexpression in HEK-293 T cells, *Ts-NBL1* and vimentin were subcloned from pDONR207 vectors to different expression vectors as described above. GST-tag or 3xFlag-tag recombination constructions were transfected in 6-well plates at 2 × 10^6^ cells per well. Transfection was performed with jetPRIME® (Polyplus-transfection) according to manufacturer’s instructions.

### Yeast two-hybrid screening procedure

The yeast two-hybrid procedure was performed according to a previously used protocol (Caignard et al. 2007). Briefly, *Ts-NBL1*-FL, *Ts-NBL1*-N, and *Ts-NBL1*-C were transferred into the Gal4-BD yeast two-hybrid vector pPC97 (Invitrogen) from pDONR207 and transformed in Y2H gold yeast strain (Clontech) according to standard procedures. The GAL4-BD-*Ts-*NBL1-FL, -Nter, and -Cter fusion proteins were tested for autonomous transactivation of *HIS3* reporter gene and then used to screen by mating a human lung epithelial cell cDNA library (derived from A549) cloned into pDEST22 vector (Invitrogen) and already established in Y187 yeast strain (Clontech). After growing on a selective medium lacking histidine and supplemented with 80 mM 3-amino-triazole (Sigma-Aldrich), [His +] colonies were picked and maintained for extra 3 weeks to eliminate false positives. AD-cDNAs from [His +] colonies were amplified by PCR using primers that hybridize within the pDEST22 regions flanking cDNA inserts. PCR products were sequenced and analyzed by blast to identify the host proteins interacting with *Ts-*NBL1.

### Co-affinity purification experiments

HEK-293 T cells (kindly provided by Dr. G. Caignard) were plated in 6-well plates (2 × 10^6^ cells per well) and 24 h later transfected with 500 ng of GST-tagged vectors and 300 ng 3xFlag-tagged vectors. Two days after transfection, HEK-293 T cells were washed in phosphate-buffered saline (PBS), and then resuspended in lysis buffer (20 mM MOPS-KOH pH 7.4, 120 mM of KCl, 0.5% Igepal, 2 mM β-Mercaptoethanol), supplemented with Complete Protease Inhibitor Cocktail (Roche). Cell lysates were incubated on ice for 20 min, and then clarified by centrifugation at 15 000 g for 15 min. For pull-down analysis, 300 µl of each protein extraction was incubated for 2 h at 4° C with 35 µl of glutathione-sepharose beads (Amersham Biosciences) to purify GST-tagged proteins. Beads were washed 3 times in ice-cold lysis buffer and proteins were recovered by boiling in denaturing loading buffer (Invitrogen).

### Western blot analysis

The purified complexes and protein extracts were analyzed by 12% SDS–polyacrylamide gel electrophoresis (SDS-PAGE) and transferred on a nitrocellulose membrane. Proteins were detected using standard immunoblotting techniques. GST-tagged proteins were detected with a rabbit polyclonal anti-GST antibody (Sigma-Aldrich) and a HRP-conjugated anti-rabbit secondary antibody (Invitrogen). 3xFlag-tagged proteins were detected with a mouse monoclonal HRP-conjugated anti-3xFlag antibody (Sigma-Aldrich).

### Quantitative PCR analysis

HEK-293 T cells were plated in 24-well plates (2 × 10^5^ cells per well). Twenty-four hours later, 0.5 µg/well pCIneo-3xFlag vector alone or fused with *Ts-NBL1* was transfected with jetPRIME® (Polyplus transfection). Two days after transfection, cells were recovered in PBS and total RNA was isolated by RNAqueous®-Micro Kit (Invitrogen) according to manufacturer’s instructions. RNA yields were evaluated using a NanoDrop spectrophotometer. cDNA synthesis was achieved by starting from 2 µg of total RNA following manufacturer’s recommendation. Quantitative PCR was performed to measure transcription levels for a 100 bp vimentin fragment (primers: Fwd: 5′-AGGCAAAGCAGGAGTCCACTGA-3′; Rev: 5′-ATCTGGCGTTCCAGGGACTCAT-3′) and a 131 bp fragment of the GAPDH housekeeping gene (primers: Fwd: 5′-GTCTCCTCTGACTTCAACAGCG-3′; Rev: 5′-ACCACCCTGTTGCTGTAGCCAA-3′) on 0.5 µl of cDNA synthesis reaction mix using the Luminaris Color HiGreen Master Mix (Thermo Fisher) in a LightCycler 480 (Roche Diagnostics).The PCR cycle was an initial incubation of 10 min at 95 °C, 40 amplification cycles of 10 s at 95 °C, of 10 s at 60 °C, and of 30 s at 72 °C, during which SYBR Green fluorescence levels were collected. Results were normalized using expression levels of the housekeeping gene.

### Immunostaining and subcellular localization

To perform subcellular localization experiments, 12-well plates containing coverslips were seeded with 1 × 10^5^ C2C12 cells per well. Twenty-four hours later, Cherry alone and Cherry-*Ts-NBL1* vectors were transfected by jetPRIME® following manufacturer’s recommendation. Twenty-four hours after transfection, cells were incubated with 4% PBS-PFA for 20 min, and then washed 2 times with PBS and incubated for 45 min at 37 °C with blocking solution (5%BSA, 0.3%Triton® X-100). After removing blocking solution, cells were incubated with rabbit polyclonal anti-vimentin antibody (Invitrogen), washed and stained with anti-rabbit Alexa Fluor-488-conjugated secondary antibody (Invitrogen). Finally, cells were incubated for 5 min in a PBS containing DAPI (4′-6-Diamidino-2-phenylindole) at 10 µg/ml and covered with aqueous mounting medium. Image acquisitions were performed using a Leica DMI 8 confocal microscope.

### Statistical analysis

All the data were shown as arithmetic means ± standard deviation (SD) and performed with Kruskal–Wallis test followed by Dunn’s post-test. The statistical test was regarded significant as follows: ∗ *p* < 0.05; ∗  ∗ *p* < 0.01.

## Results

### Phylogenic analysis and sequence alignments of *Ts-*NBL1

**Fig. 2:**
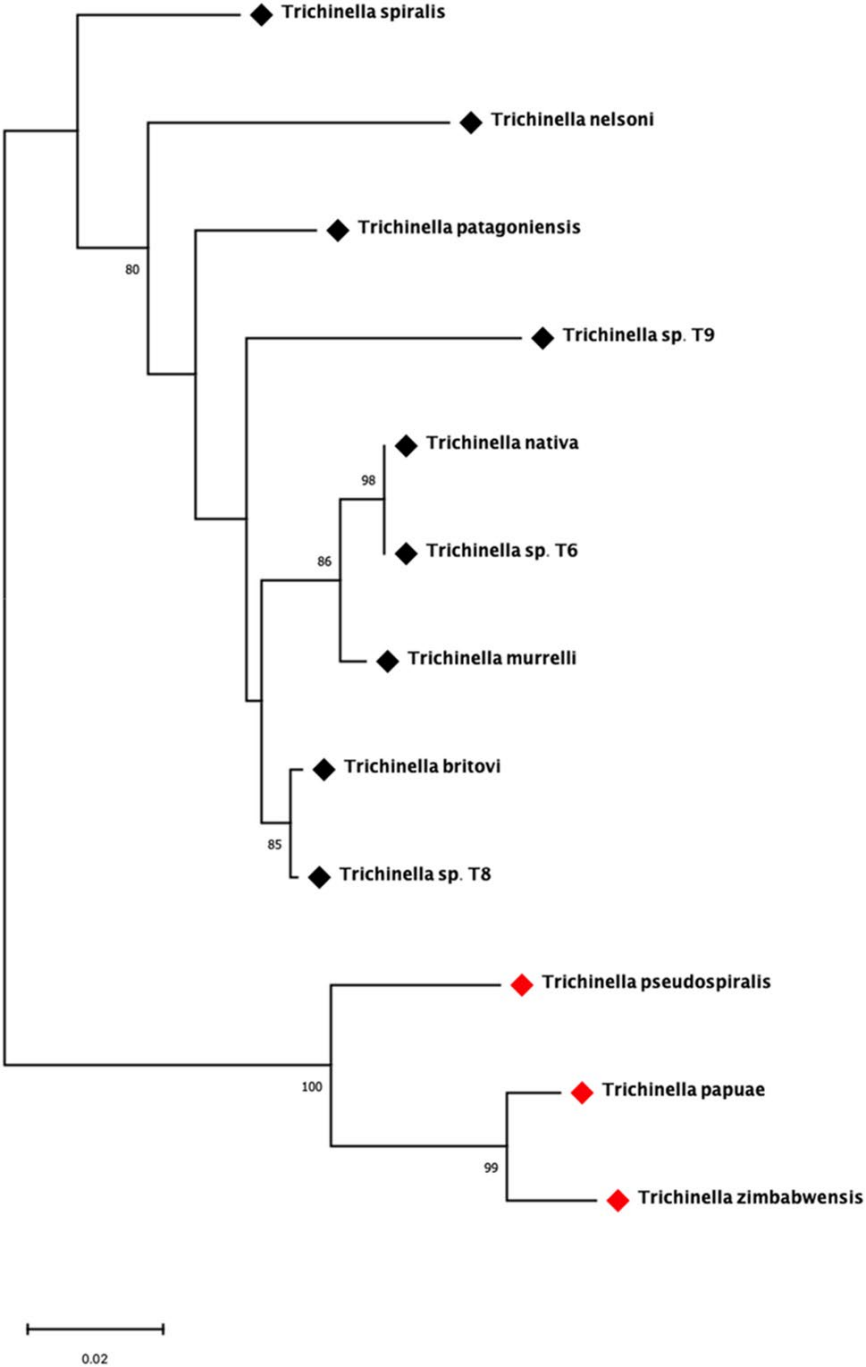
Phylogenetic tree constructed by MEGA. The maximum likelihood phylogenetic tree of NBL1 from 9 species and 3 genotypes generated in MEGA. Bootstrap values which are higher than 80 are indicated on branches. Black diamonds indicate *Trichinella* encapsulated species, red diamonds indicate *Trichinella* non-encapsulated species

**Fig. 3:**
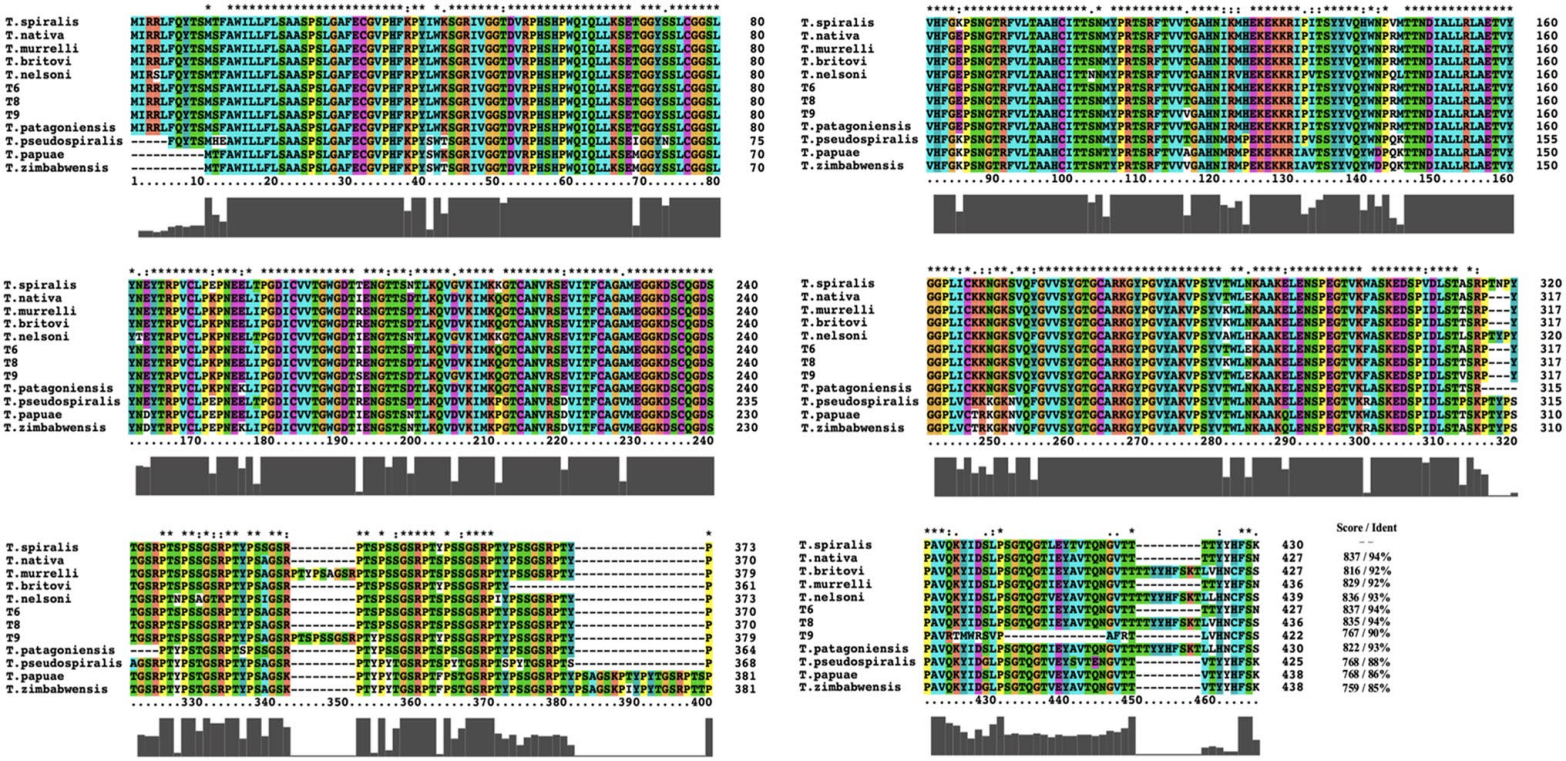
Sequence alignment of *Ts-*NBL1 from *Trichinella spiralis* with other species within the *Trichinella* genus. The multiple sequences alignment were performed in the MEGA and ClustalX, the following symbols donating the degree of conservation in each column: * fully conserved residue, conservative substitution, and semi-conservative substitution. Gray qualify curve indicated that residues identical to *Ts-*NBL1. The numbers at the end of each sequence represent the score and percent identity to *Ts-*NBL1

As shown in the phylogenetic tree generated with *Ts-*NBL1 and its orthologues (Fig. 2), the sequences are grouped as the two branches corresponding to on one side the encapsulated and on the other side the non-encapsulated *Trichinella* species. Compared with encapsulated clade (black squares), the species in non-encapsulated (red squares) are a monophyletic group with bootstrap value of 100. Also, a homology comparison of *Ts-*NBL1 and other orthologues in the genus *Trichinella* was determined among these sequences (Fig. 3). Interestingly, *Ts-*NBL1 homologous genes show different identities between encapsulated and non-encapsulated species. In encapsulated species (*T. nativa, T. britovi*, *T. murrelli*, *T. nelsoni*, *T. patagoniensis*, and genotype T6, T8, T9), there are more than 90% identity with *Ts-*NBL1, while with non-encapsulated species *T. pseudospiralis*, *T. papuae*, and *T. zimbabwensis*, the identity drop to 88%, 86%, and 85% respectively.

### Y2H screening of *Ts-*NBL1 potential interacting proteins

To screen the host proteins interacting with *Ts-*NBL1, Y187 yeast cells expressing a human cDNA library were used to mate with Y2H gold yeast strain expressing *Ts-*NBL1 fragments, as described above. After 6 days of culture on selective medium, [His +] colonies were picked, purified during 3 weeks by culturing on selective medium. A total of 193 cultured colonies allowed identifying *Ts-*NBL1-C binding partners after elimination of false positives (Fig. S1a). These 193 colonies were amplified by PCR from zymolase-treated yeast colonies using primers that hybridize within the pDEST22 regions flanking cDNA inserts (Fig. S1b). PCR products were sequenced, and cellular interactors were identified by blast analysis. A total of 193 colonies corresponded to 20 potential interacting proteins are listed in this table according to the number of colonies from most to least (Table 2). Thirty-one colonies corresponded to undescribed proteins, which are not shown in this table. Gene ontology analysis indicated that these interactants participate in putative signaling pathway and biological processes (Fig. S2), such as participating in caspase cascade in apoptosis (VIM, KRT18, LMNB1), striated muscle contraction (VIM), positive regulation of cell cycle G2/M phase transition (NPM1), and positive regulation of collagen biosynthetic process (VIM). The analysis of the function, combined with the number of positive colonies, led to the consideration of vimentin as an important interactant.

**Table 2:** *Ts-*NBL1 potential interacting proteins screened by Y2H

Protein names	Abb	Accession N°	Colonies
Endogenous Retrovirus Group K3 Member 1	ERVK3-1	ENSP00000489180	56
Potassium Channel Tetramerization Domain Containing 10	KCTD10	ENSP00000441672	29
Vimentin	VIM	ENSP00000435613	25
Mitochondrial Assembly Of Ribosomal Large Subunit 1	MALSU1	ENSP00000419370	10
Nucleophosmin	NPM1	ENSP00000377408	9
EF-hand Calcium Binding Domain 14	EFCAB14	ENSP00000361001	5
KIAA1549 Like	KIAA1549L	ENSP00000433481	4
Kinesin Family Member C3	KIFC3	ENSP00000458009	4
Diacylglycerol Kinase Delta	DGKD	ENSP00000386455	3
Envoplakin	EVPL	ENSP00000301607	3
E1A Binding Protein P400	EP400	ENSP00000331737	2
Heterogeneous Nuclear Ribonucleoprotein U Like 1	HNRNPUL1	ENSP00000473178	2
Keratin 18	KRT18	ENSP00000447278	2
Lamin B1	LMNB1	ENSP00000378761	2
Caspase 8 Associated Protein 2	CASP8AP2	ENSP00000478179	1
Potassium Channel Tetramerization Domain Containing 11	KCTD11	ENSP00000328352	1
Keratin 8	KRT8	ENSP00000447881	1
MIF4G Domain Containing	MIF4GD	ENSP00000484245	1
Ribosomal Protein L39	RPL39	ENSP00000355315	1
Tripartite Motif Containing 23	TRIM23	ENSP00000274327	1

### *Ts-*NBL1 interacts with vimentin

**Fig. 4:**
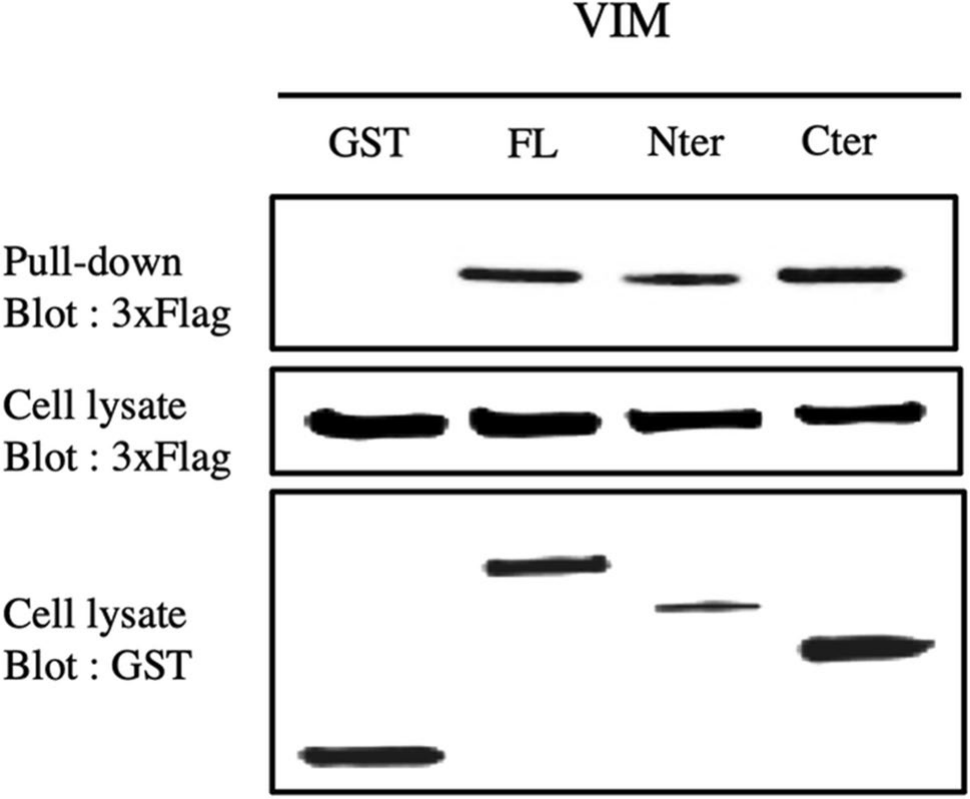
Pull-down of interaction between *Ts-*NBL1 and vimentin. HEK-293 T cells were co-transfected with expression plasmidic vectors encoding GST alone or fused to *Ts-*NBL1 (500 ng/well) and encoding 3xFlag expression vector fused to vimentin (300 ng/well). Cell lysates from transfected cells were collected at 48 h post-transfection. Then pull-down using glutathione-sepharose beads assayed protein interactions complexes. GST- and 3xFlag- tag were detected by immunoblotting

To verify the interactions between *Ts-*NBL1 and vimentin in mammalian cells, *Ts-*NBL1 fused with GST-tag and vimentin fused with 3xFlag-tag were co-expressed in HEK-293 T cells and purified with glutathione-sepharose beads. Expression of each of these proteins in HEK-293 T cells was confirmed by detecting different expression tags in cell lysates. Pull-down samples were collected and detected 3xFlag-tag to assess protein interactions. Since negative control GST vector alone did not show detectable products (Fig. 4), these results indicated that vimentin was able to interact with *Ts-*NBL1.

### *Ts-*NBL1 up-regulates vimentin mRNA expression

**Fig. 5:**
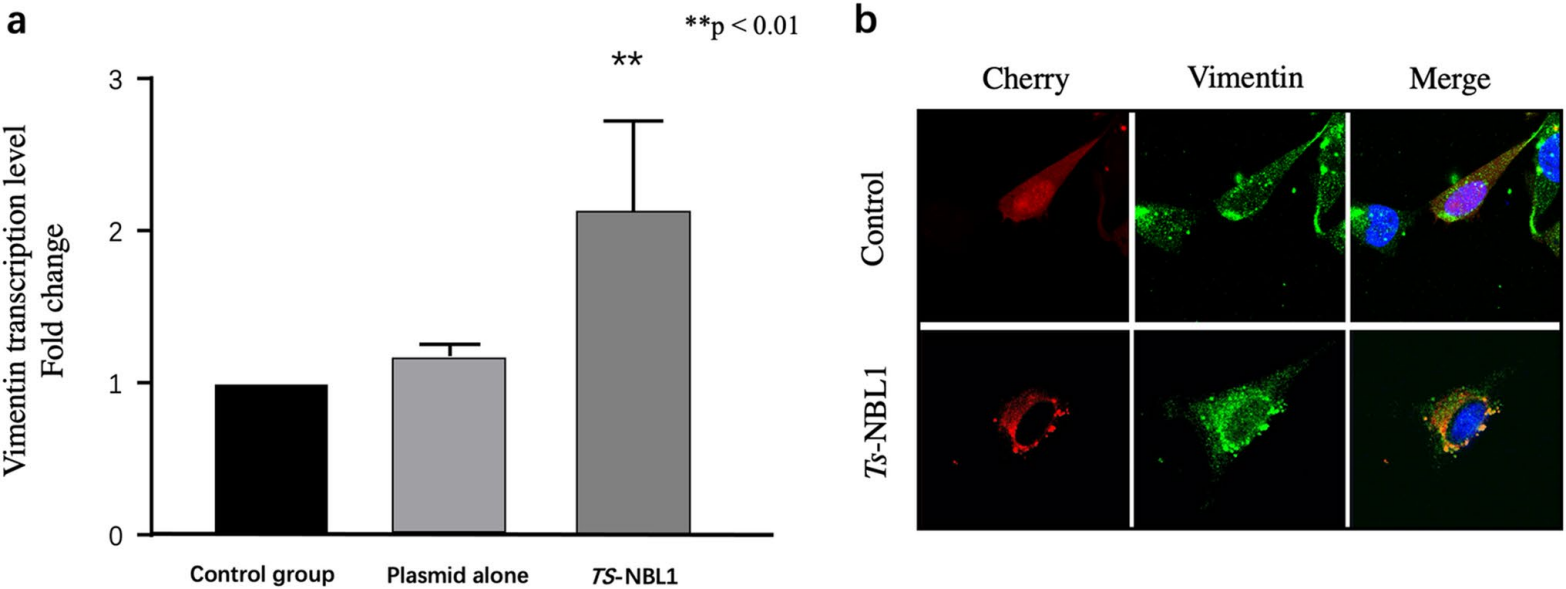
Transcription level of vimentin and subcellular localization after *Ts-*NBL1 transfection. **a** Transcription level of vimentin after *Ts-*NBL1 transfection. pCIneo-3xFlag plasmidic vector alone or encoding *Ts-*NBL1 was transfected in HEK-293 T cells (500 ng/well), and transcription level of vimentin was quantified by qPCR. Data were normalized and results are shown as the mean ± SD for at least three independent experiments for each group. Significant differences is as **p < 0.01. **b** Immunostaining and subcellular localization of *Ts-*NBL1 and/or vimentin in C2C12 cells. C2C12 cells were transfected with 1 µg of each plasmid encoding Cherry alone or fused to *Ts-*NBL1. Green color corresponds to vimentin whereas red corresponds to Cherry / *Ts-*NBL1 proteins

In order to determine the effect of *Ts-*NBL1 on vimentin expression, *Ts-*NBL1 was over expressed in HEK-293 T cells to detect vimentin mRNA expression. After normalizing with GAPDH expression, vimentin transcription level was evaluated. Compared to the control group, *Ts-*NBL1 transfection significantly increased the expression of vimentin with a twofold increase (Fig. 5a).

### Immunostaining and subcellular co-localization

To identify the subcellular localization of *Ts-*NBL1 in cells and whether it co-localized with vimentin, *Ts-*NBL1 was expressed in C2C12 cells by reconstruction Cherry plasmids transfection (Fig. 5b). Vimentin was distributed around the cell nucleus and radiated towards the plasma membrane, participating in the cytoskeleton without any cell changes. *Ts-*NBL1 exhibited a perinuclear distribution radiating towards the periphery of the cytoplasm. A co-location of *Ts-*NBL1 and vimentin was demonstrated, as both signals have fused the same cell locations.

## Discussion

It was important to compare the genomic sequences of NBL1 between the two different clades of *Trichinella* (encapsulated *versus* non-encapsulated) as this protein, which is expressed at early stage of development, seems to be essential for understanding the mechanisms of interaction of this parasite with its host and in particular in the establishment of the parasitic nurse cell. In this way, the comparative study of the sequences shown the evolution of the sequences between the different species of the genus *Trichinella*. A phylogenic analysis of *Trichinella* has shown that encapsulated and non-encapsulated *Trichinella* taxa diverged from their most recent common ancestor ∼21 million years ago, with taxon diversifications happening ∼10 − 7 million years ago (Korhonen et al. 2016). Interestingly, the NBL1 sequences are also organized into two groups where encapsulated and non-encapsulated *Trichinella* species are distributed. A strong link is thus confirmed between the NBL1 sequences and the membership of one of the two clades. Differences between encapsulated and non-encapsulated species could explain some differences on the nurse cell (NC) formation like the thick/thin collagen expressed surrounding the cell. Unveiling the function of *Ts-*NBL1 could hold one of the key to understand the interactions of *Trichinella* with its host muscle cell and its differentiation as nurse cell structure.

In the present study, Y2H screening was used to identify host proteins interacting with *Ts-*NBL1 to understand the molecular and/or cellular mechanisms by which cellular pathways *Ts-*NBL1 works in the cell. A total of 193 interacting colonies corresponding to 20 proteins from a human cDNA library were identified as potential interacting proteins of C-terminal part of *Ts-*NBL1. These potential interacting proteins are involved in many of the same biological pathways, such as the apoptotic process, cell proliferation, cell differentiation, and signal transduction. Among these potential interactants, we focused on vimentin, which was found in a large number of colonies. Vimentin is the major intermediate filaments protein found in mesenchymal cells (Eriksson et al. 2009) and also as a marker of regeneration in muscle cells (Gallanti et al. 1992). Moreover, growing evidence suggests that vimentin is involved in a series of metabolic, signaling, and regulatory processes that are not related solely to mechanical function (Ivaska et al. 2007; Kwak et al. 2012; Zhang and Stefanovic 2016).

For the first time, our study identified the reliable interaction between vimentin and *Ts-*NBL1 by co-affinity assay in vitro. Moreover, this interaction was confirmed by GST pull-down approach. Immunofluorescence results indicated that *Ts-*NBL1 displayed high intensity, around the nucleus with a co-localization with vimentin, at the cytoplasmic level. Many cellular signaling pathways are altered in various physiological conditions, especially reactions involving proteases (Singhirunnusorn et al. 2005). Although we are currently unable to determine whether this interaction was related to the serine protease activity of *Ts*-NBL1, the model we use was to study the co-localization of *Ts*-NBL1 at the 24-h fixed point of transfection. It could be further captured by a lifetime image instead of at a fixed point to figure out how long the interaction lasts or whether the process of interaction is transient.

The transfection of *Ts-*NBL1 significantly upregulated the expression of vimentin with a twofold increase. This major result in the knowledge of *Trichinella* interaction with its host cells could explain the impact on collagen expression for the nurse cells construction. Indeed, studies have shown that vimentin interacts with LARP6, which is involved in the regulation of collagen to stabilize collagen mRNA. The stabilization of collagen mRNA is the main mechanism of high collagen expression. Vimentin knockout fibroblasts produce reduced amounts of type I collagen due to decreased stability of collagen mRNA (Challa and Branko 2011). On the other hand, newborn larvae invade muscle cells and can cause mechanical damage and induce collagen production in surrounding cells, which accumulates in response to tissue injury. Vimentin is required for this accumulation and have been reported to mediate the early signaling process of tissue repair (Cheng et al. 2016; LeBert et al. 2018). We proposed that vimentin contributes to the collagen synthesis in the infected cells. We demonstrated that vimentin binds to *Ts-*NBL1 and also contributes to *Ts-*NBL1-dependent upregulation of vimentin, which may affect accumulation of collagen around nurse cell.

Vimentin is a marker in the process of muscle cell regeneration (Bornemann and Schmalbruch 1992). Vimentin and the muscle-specific protein desmin belong to the same assembly group and exhibit a rather high amino acid sequence identity (Bär et al. 2004). While desmin is only expressed at low levels during the early stage of muscle cell development, it increases as the cell approaches terminal differentiation. In other words, vimentin was detected in early developing muscle, but not in fully differentiated muscle (Vater et al. 1994; Vaittinen et al. 2001; Güttsches et al. 2015). However, the muscle cells infected by *T. spiralis* undergo dedifferentiation at the beginning, which provides more chance for the interaction of *Ts*-NBL1 and vimentin. It seems like a strategy of the parasite in this process: *Ts*-NBL1 interacts with vimentin even upregulating the expression of vimentin to promote the cell’s destined fate to re-differentiate into NC. It has been reported that *Trichinella* can significantly upregulate vimentin gene expression in muscle cells 23 dpi, which is more noticeable with *T. spiralis* than *T. pseudospiralis* (Wu et al. 2008). This may be due to the differences in NC formation induced by *T. spiralis* (encapsulated) and *T. pseudospiralis* (non-encapsulated). NC formation in *T. spiralis* involves the fusion of transformed infected muscle cells and misdifferentiated satellite cells, whereas in *T. pseudospiralis*, the satellite cells are activated but never fuse to the nurse cell cytoplasm, resulting in incomplete cysts formation (Wu et al. 2001; Boonmars et al. 2004). *Ts-*NBL1 could thus upregulate vimentin expression in non-muscle cells, and whether the same phenomenon occurs in muscle cells remains to be confirmed. As we have an ethical duty to reduce the use of animal experiments, ex vivo model allowing the parasitic invasion of differentiated muscle cells will be needed to further identify the interaction that occurs in the actual infection of *Trichinella*.

Our research described for the first time the interaction of a *T. spiralis* stage-specific protein with a protein of the cell cytoskeleton. It is thus proposed that *T. spiralis* uses NBL1 as a secreted signaling molecules to carry out its own developmental programs like all the mammalian intercellular communication systems (Despommier 1998). In this way, NBL1 could be considered “parakines.” Our study would thus be the first identification and characterization of the communication between the mammalian cell and the worm during nurse cell formation. The importance of this interaction *Ts-*NBL1 /vimentin is all the more important as the parasitic protein is expressed at an early and invasive stage of the parasite’s live cycle, which coincides with the installation in the muscle cell. An overexpression of vimentin could explain a collagen production mechanism that contributes to the constitution of a NC with a protective collagen capsule as described for encapsulated species.

In summary, the identification of key *Ts*-NBL1 protein interactions in the early stage of *T. spiralis* provides insight into the molecular and cellular mechanisms involved in *Trichinella* survival in host cells. This contributes to the understanding of nurse cell formation and high collagen expression in the encapsulated clade.

## Supplementary Information

Below is the link to the electronic supplementary material.

Supplementary file1 (DOCX 1633 KB)

## Abbreviations

NC: Nurse cell
dpi: Days post-infection
NBL: Newborn larvae
*T. spiralis*: *Trichinella spiralis*
*Ts-*NBL1: First *Trichinella spiralis* newborn larvae stage serine protease-like protein
*Ts-NBL1*: Italic type, represents the gene
Y2H: Yeast two-hybrid
