# Supplementary figures and images for: A Trichinella spiralis new born larvae-specific protein, Ts-NBL1, interacts with host’s cell vimentin
Source: Parasitol Res. 2022 Mar 23;121(5):1369–78. doi: 10.1007/s00436-022-07479-7 (PMC8993751; doi:10.1007/s00436-022-07479-7)

Fig. S1


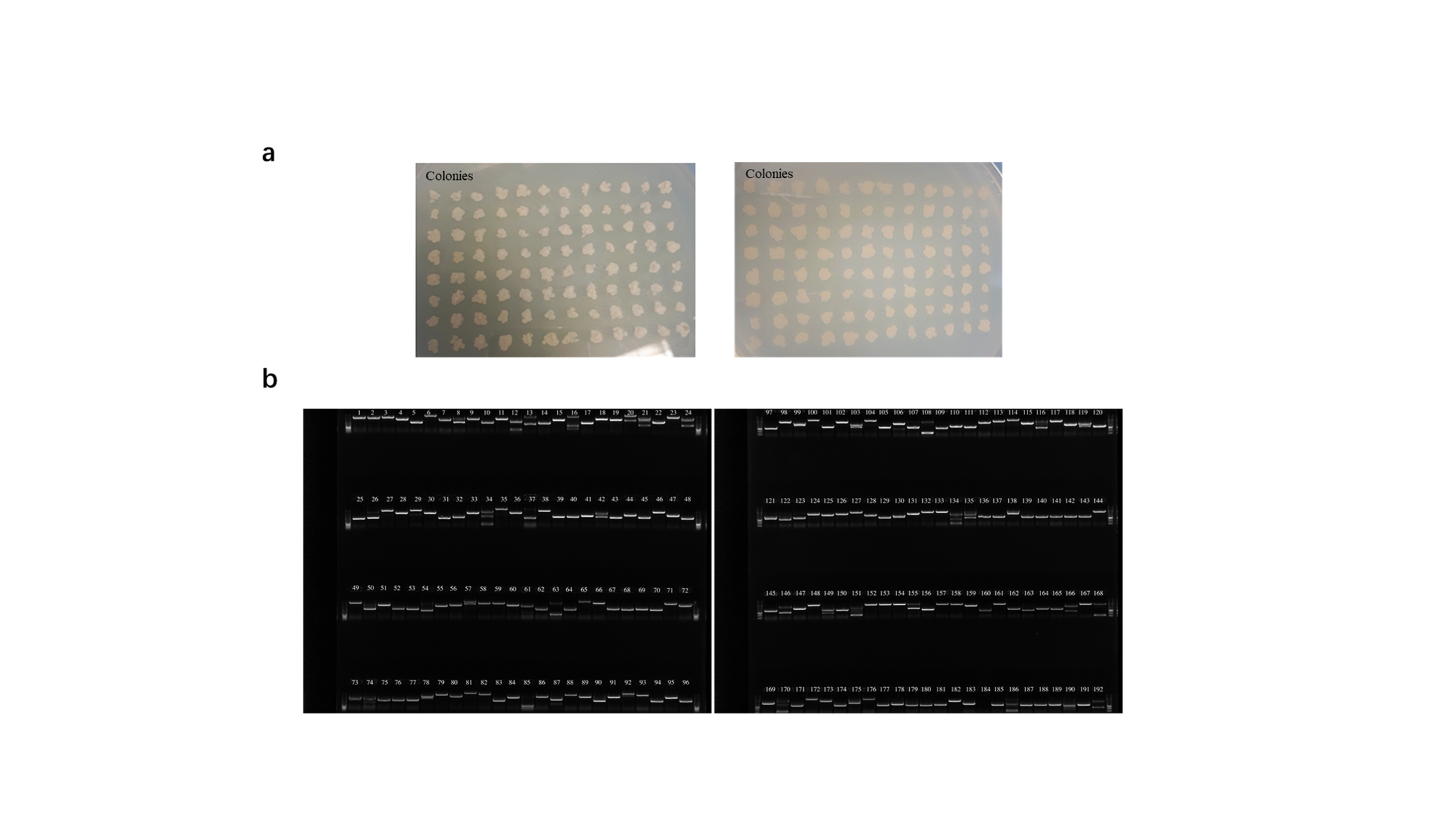


Fig. S2


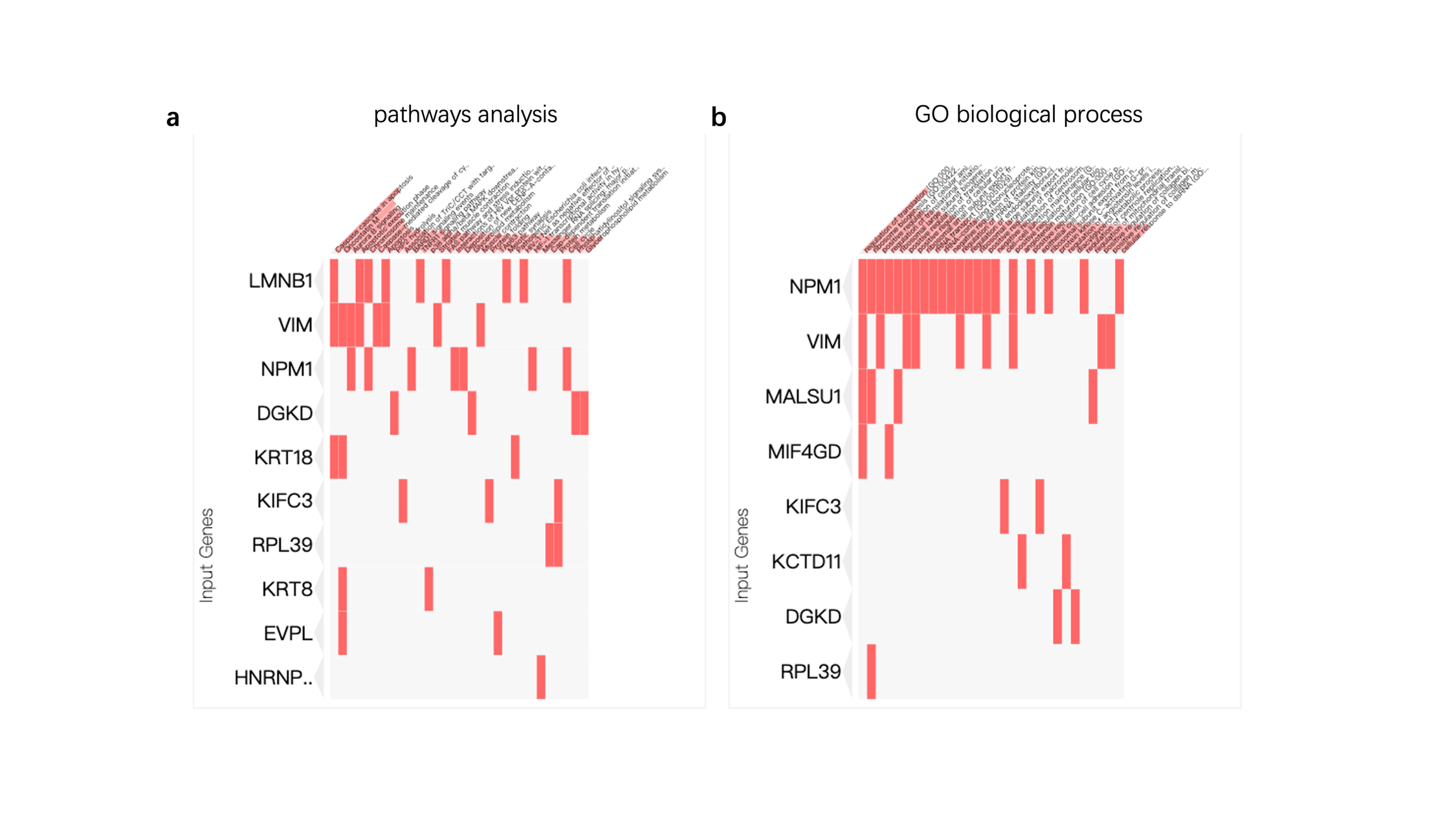

Supplement: Supplementary file 1 — Supplementary file1 (DOCX 1633 KB) [file 436_2022_7479_MOESM1_ESM.docx]
